# Cryosurgery, Intralesional Methotrexate and Imiquimod for Keratoacanthoma: Tuning the Combination

**DOI:** 10.1155/2019/3489748

**Published:** 2019-11-11

**Authors:** Georgios Gaitanis, Ioannis D. Bassukas

**Affiliations:** Department of Skin and Venereal Diseases, Faculty of Medicine, School of Health Sciences, University of Ioannina, Ioannina, Greece

## Abstract

Keratoacanthomas (KA) are self-regressing, destructively expanding keratinocyte skin neoplasms typically characterized by sudden onset of explosive growth followed by complete involution. Cryosurgery, intralesional methotrexate and imiquimod have been used alone or in combination of two for the treatment of KA. Presently 3 patients (49, 60, and 65 years old; two females, one suspected with Ferguson-Smith syndrome), with 5 KA (6–24 mm maximal diameter) were treated with the combination of cryosurgery (liquid N_2_, open spray, 2 cycles of 15 sec each) and intralesional methotrexate (2.5–30 mg cumulative dose) and subsequent daily application of imiquimod (14–35 days). Starting with 4 cryosurgery/intralesional methotrexate sessions and 5 weeks daily imiquimod, to document feasibility and efficacy we progressively reduced the intensity of the treatment to one cryosurgery/intralesional methotrexate (total dose: 5 mg) session and 14 days of daily imiquimod without compromising efficacy. KA stopped growing promptly with sustained clearance after 6–24 months follow up, implicating a huge potential of therapeutic synergy of the employed modalities in the management of KA. We suggest that, optimized, the present three modalities combination (one session mild cryosurgery/low dose, 5 mg intralesional methotrexate and 2 weeks once daily imiquimod) is a promising treatment for KA that merits evaluation in further studies.

## 1. Introduction

Keratoacanthoma (KA) is a distinct subset of self-regressing, locally invasive and destructively expanding keratinocyte skin neoplasms with squamous cell carcinoma micromorphology. KA are characterized by the sudden onset of an explosive, though limited growth phase followed by complete tumor tissue involution [1]. However, the evolution process of any particular KA is somehow unpredictable and few cases may acquire large dimensions that can eventually result in dysmorphic, potentially mutilating scars. This coupled with the possibility of missing a frank squamous cell carcinoma (SCC) prompts therapeutic intervention for all patients with this neoplasm [1]. Furthermore, multiple KA may appear in younger ages within the framework of syndromic KA forms, like the Ferguson-Smith syndrome. More recent interest for novel treatment modalities for KA has revived as KA emerges as an important toxicity event of the vemurafenib [2] and probably also of the pembrolizumab [3] treatment in patients with advanced malignant melanoma.

In the present retrospective study we describe the successful treatment of KA with the minimally invasive combination of concomitant sessions of cryosurgery and intralesional methotrexate and cycles of once-daily imiquimod application starting at the same evening of the cryosurgery-methotrexate treatment day. Initially, we evaluated the feasibility of the combination treatment protocol with an intensive plan of weekly repetitions of cryosurgery-methotrexate (moderate high dose) sessions during a 5-week once daily imiquimod application cycle. In the following, we optioned a step-wise up-down adaptation of the intensity of all three composing modalities, to optimize the feasibility-efficacy relationship of the proposed combination. Aim of this explorative study was to enhance the selectivity of the present triple combination modality for KA by (a) documenting feasibility and (b) adapting the intensity of all combined modalities to optimize efficacy.

## 2. Case Reports

Single KA were biopsied prior to the intervention, while in the case of multiple KA treated in parallel, only one per treatment session was biopsied. Monitoring of methotrexate was performed according to corresponding treatment guidelines [4].

**Figure 1:**
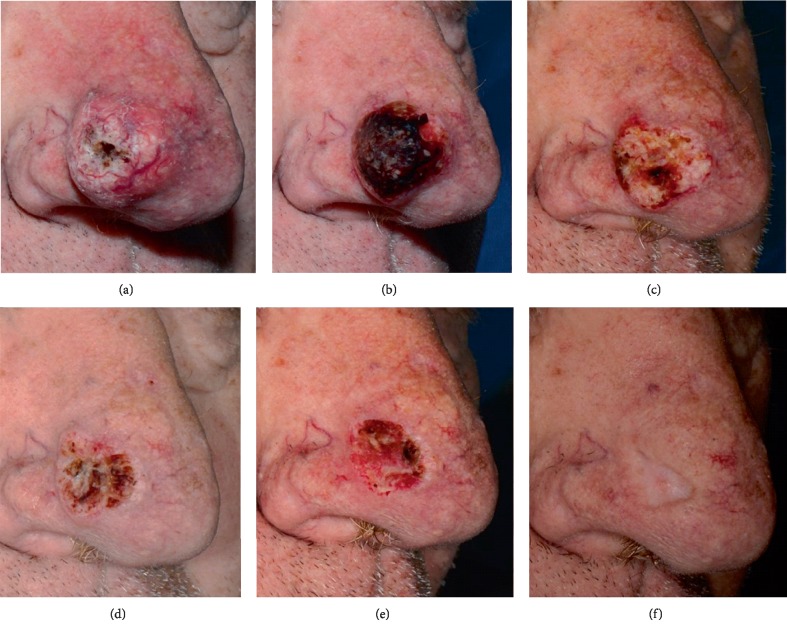
Patient 1 with a 24 mm in maximal diameter keratoacanthoma. The patient was treated with weekly sessions of cryosurgery (liquid N_2_, open spray, 2 cycles of 15 sec each), intralesional methotrexate (7.5 mg/weekly sessions) (Panels a–d) and daily application of imiquimod. Panel a: The tumor at baseline. Panels b–d: The tumor at weekly intervals at the days of cryosurgery and intralesional methotrexate. Panel e: Gradual tumor involution is achieved and the patient is treated with daily imiquimod for another week (35 days imiquimod in total). Panel f: The tumor has cleared and a deep scar has remained, representing the “tumor ghost”, i.e., the area destroyed by the rapidly expanding keratoacanthoma. Photographs were acquired with modification of a previously used equipment [20] employing a Nikon D610 camera with a spatial resolution of 6016 × 4016 pixels. A 60 mm prime lens was adapted with a vertical polarized filter and a SIGMA EM-140 Macro ring flash with an additional vertical polarized filter. The photographs were taken with the axons of polarization of the two filters in vertical position to each other for cross-polarization to enhance the visualization of the vascular bed [21].

*Patient 1* (Figure 1). A 65-year old man with unremarkable medical history presented with a rapidly enlarging tumor on the nose that within one month acquired 24 mm maximal diameter in size. Merging the clinical features and the findings of a punch biopsy we diagnosed KA. Once weekly cycles of concomitant cryosurgery (liquid N_2_, open spray, 2 cycles of 15 sec each) and intralesional methotrexate (7.5 mg per session) were initiated in parallel to once daily 5% imiquimod cream application locally to the KA and a 0.5 cm wide skin rim around it for a total of 35 days. Four sessions of cryosurgery and intralesional methotrexate (total dose: 30 mg) were applied. The KA regressed rapidly, leaving a shallow scar corresponding approximately to the area destructed by the tumor during growth. The patient was relapse-free at the 24-month evaluation.

*Patient 2*. A 60-year old female presented with a KA of 2 weeks duration at the left mandible, 10 mm in maximal diameter. Biopsy, cryosurgery (liquid N_2_, open spray, 2 cycles of 15 sec each) and intralesional methotrexate (5 mg) were provided in one session and once daily imiquimod application for 5 weeks was started. The lesion rapidly regressed and the patient is relapse free for 24 months, with a hypopigmented scar at the site of treatment.

**Figure 2:**
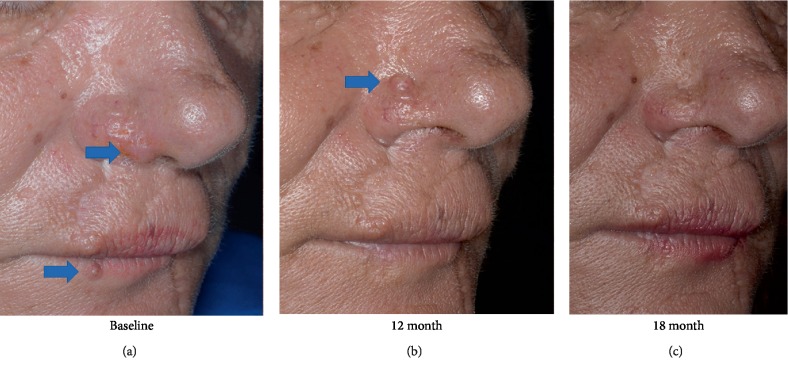
Patient 3. Panel a: The patient at presentation with two keratoacantoma lesions (arrays). Scars from previous surgeries for the same diagnosis are evident. Panel b: At 12 months follow-up the two lesions in complete remission with a swallow scar. Meanwhile a new lesion has appeared (arrow). Panel c: The patient at the 18 months follow-up. All photographs are taken with parallel polarization to increase the perception of skin relief [21]. For details of photography see Figure 1.

*Patient 3* (Figure 2). A 49-year old female with a history of 8 resected KA from the age of 28 presented with 2 new KA lesions, on the nose and the lower lip (8 mm and 6 mm in maximal diameters, respectively). Her medical history was otherwise unremarkable and in the family no KA cases could be recorded. Ferguson-Smith syndrome was suspected [1]; however, the patient did not consent for additional screening. The KA were treated with cryosurgery (liquid N_2_, open spray, 2 cycles of 15 sec), intralesional methotrexate (2.5 mg/lesion) and daily application of imiquimod for 14 days. The shorter scheme was selected based on the observation that lesions rapidly involuted in the aforementioned Patients 1 and 2. At 12 months follow-up, a new lesion (8 mm maximal diameter) at the left side of the nose was treated likewise. All treated lesions regressed promptly and remained in complete remission, at 24 months follow-up, from her initial presentation.

## 3. Discussion

Experts still advocate surgery (conventional or Mohs'), if feasible, as the first line modality to manage KA [1]. As a main argument discussed in favor of this proposal is the traditionally quite subjective approach to the KA diagnosis [5], resulting in uncertainty to differentiate KA from SCC, at least in an appreciable subset of cases [1]. However, recent advances in the pathophysiology of KA clearly underline its unique nature, provide molecular evidence to differentiate it from SCC and have substantially sharpened our clinical-laboratory instruments to distinguish between the two conditions at the patient's level [6, 7]. Consequently, these latter developments call for reevaluation of the therapeutic priorities to approach the treatment of KA, by optimally addressing the benign biological behavior on one hand and the unpredictable tissue-destructive potential of this self-limited, skin growth on the other. Accordingly, in order to successfully substitute surgery as the mainstream modality, any proposed alternative modality should fulfill the same achievements, i.e., (a) abrupt growth stop as typical for surgical lesion removal, (b) sustained clearance of the treated lesion, and (c) minimal risk of KA induction in the surrounding skin areas by the therapeutic intervention itself. It is worth noting that, if effective, minimally invasive modalities are more efficacious in the management of patients with multiple KA lesions.

To date, the available nonsurgical modalities, employed alone, are of somewhat inferior effectiveness compared to surgery [1]. A way to improve effectiveness of these latter modalities is to evaluate rational combinations of them for synergy to enhance selectivity [8]. Herein we reported the preliminary results of an exploratory, minimally invasive approach to the treatment of KA with a fixed timing combination of three modalities, i.e., cryosurgery, intralesional methotrexate and imiquimod. All three are widely available modalities and were selected to be studied in a designed combination based on their accepted efficacy for KA, however, in adequately high treatment intensities, and also because they are not known to possess antagonistic therapeutic potential to each other. They have been applied with appreciable success either alone [9, 10] or in certain combinations of two [11] to treat KA. Our main aim was through a fixed combination to minimize the intensity of each consisting modality whereas optimizing the efficacy of the treatment. Intralesional MTX was collected as the backbone of the present combination, as it is the most preferable drug reported in the literature for the intralesional treatment of KA due to sufficient effectiveness (overall cure rate: 91%) [12], quite moderate costs per procedure and convenience of application (less pain compared e.g., to intralesional 5 fluorouracil) [13]. Mean total doses of 38.2 mg MTX in an average of 2.1 injections per lesion were needed to achieve above effectiveness levels [14]. The timing of the combination was designed to concentrate the intensity of the intervention in the very beginning of the treatment cycle, i.e., in a fashion mimicking as close as possibly the surgical excision. Due to its highest toxicity potential compared to the other two combination constituents (cryosurgery and topical imiquimod), our first priority was to enquire possible reduction of the MTX dose. Based on the encouraging feasibility and efficacy outcome, with the most intensive starting treatment scheme variation (Patient 1), we subsequently limited the applied methotrexate dose to a total of 5 mg and the cryosurgery sessions to a single one, whereas we retained the “typical” for “immunocryosurgery” 5-week imiquimod application period, as established in the treatment of basal cell carcinoma [15] (Patient 2). By the way, the total methotrexate dose applied to treat this latter lesion (Patient 2) is as low as those used for hypersensivity screening in candidates for methotrexate treatment initiation [4]. Based on the observation of optimal efficacy with this latter reduced scheme in Patient 2, in the last assessment step (Patient 3) we restricted the duration of imiquimod use from 5 to 2 weeks only once daily applications with very encouraging treatment outcome, however, in overall relatively smaller tumors. For comparison, according to literature data, generally 4–11 weeks of application are needed to obtain complete remission with topical imiquimod monotherapy [1]. Also Jeon et al. [16] based on their observation of the clinical course of KA resolution after 9 to 11 weeks of imiquimod treatment proposed that for remission treatment duration as short as 5 to maximal 8 weeks might be sufficient to clear KA. It is worth noting that for tumors <1 cm in maximal diameter (the first two neoplasms of Patient 3) a total methotrexate doses of 2.5 mg was adequate to achieve prompt remission with the present combination modality. However, since a 5 mg dose is generally regarded safe we suggest the slightly higher dose (5 mg MTX) to initiate this treatment combination for all KA. We have had the opportunity to evaluate this latter combination in the treatment of the 3^rd^ KA of Patient 3 with an excellent feasibility and efficacy-outcome.

Resuming, we propose for a newly diagnosed KA, a treatment cycle starting with a moderate cryosurgery session (liquid N_2_, open spray, 2 cycles of 15 sec) immediately followed by intralesional methotrexate (5 mg) in this order and already from the first therapy day once daily topical imiquimod (5% cream) for 2 weeks. This is a significantly shorter treatment duration compared to the approximately 8 weeks required for imiquimod alone treatment for KA [16, 17], which, if confirmed in larger studies, would mean a considerable improvement of feasibility and an indication in favor of synergistic effects in the framework of the combination. Yet, from our experience with large basal cell carcinoma ([18, 19] and unpublished data) we know that the combination of imiquimod and cryosurgery is optimized with biweekly cryosurgery session repetitions. We would like to suggest that for inadequately responding KA to a single treatment cycle repetitions of the above cycle could be enquired to improve overall treatment efficacy.

The main limitation of the present proposal is the fact that it is supported by the results of a relatively limited trial. KA is a challenging diagnosis for which no unique therapeutic proposal is accepted; yet all herein combined modalities have been used as single therapeutic modalities and proved efficacious at acceptable high rates. Moreover, our observations in the present case series indicate to a clinically significant synergy potential of the combined modalities. In conclusion, we propose a two-weekly triple modality combination module for the nonsurgical therapeutic approach to KA that combines safety and efficacy, can be repeated as required to improve efficiency and could be evaluated within appropriately designed clinical trials.
